# Diagnostic value of gamma‐glutamyl transpeptidase to alkaline phosphatase ratio combined with gamma‐glutamyl transpeptidase to aspartate aminotransferase ratio and alanine aminotransferase to aspartate aminotransferase ratio in alpha‐fetoprotein‐negative hepatocellular carcinoma

**DOI:** 10.1002/cam4.4057

**Published:** 2021-06-18

**Authors:** Jiang Li, Haisu Tao, Erlei Zhang, Zhiyong Huang

**Affiliations:** ^1^ Hepatic Surgery Center Tongji Hospital Tongji Medical College Huazhong University of Science and Technology Wuhan China

**Keywords:** alanine aminotransferase to aspartate aminotransferase ratio, alpha‐fetoprotein‐negative hepatocellular carcinoma, diagnostic markers, gamma‐glutamyl transpeptidase to alkaline phosphatase ratio, gamma‐glutamyl transpeptidase to aspartate aminotransferase ratio

## Abstract

**Background:**

The purpose of the study was to evaluate the diagnostic value of gamma‐glutamyl transpeptidase to alkaline phosphatase ratio (GAPR) combined with gamma‐glutamyl transpeptidase to aspartate aminotransferase ratio (GAR) and alanine aminotransferase to aspartate aminotransferase ratio (AAR) in alpha‐fetoprotein (AFP)‐negative hepatocellular carcinoma (HCC).

**Methods:**

A total of 925 AFP‐negative patients, including 235 HCC patients, 213 chronic hepatitis (CH) patients, and 218 liver cirrhosis (LC) patients, as well as 259 healthy controls were enrolled in this study. The differences of laboratory parameters and clinical characteristics were analyzed by Mann–Whitney U or Kruskal–Wallis *H*‐test. Receiver operating characteristic (ROC) curve analysis was used to determine the diagnostic value of GAPR, GAR, and AAR in AFP‐negative HCC (AFP‐NHCC) patients.

**Results:**

GAPR, GAR, and AAR were important parameters closely related to AFP‐NHCC. The combination of GAPR, GAR, and AAR was most effective in differentiating AFP‐NHCC group from control group (AUC = 0.875), AFP‐negative CH group (AUC = 0.733), and AFP‐negative LC group (AUC = 0.713). GAPR combined with GAR and AAR exhibited a larger AUC than single ratio or pairwise combination for distinguishing AFP‐NHCC group with TNMⅠstage, BCLC stage A, and tumor size less than 3 cm. The diagnostic value of GAPR combined with GAR and AAR was higher in AFP‐NHCC and was also reflected in the TNM stage, Barcelona Clinic Liver Cancer (BCLC) stage and tumor size.

**Conclusions:**

GAPR combined with GAR and AAR were effective diagnostic markers of AFP‐NHCC, especially in patients with good liver function, early stage or small size.

## Graphical abstract

Gamma‐glutamyl transpeptidase to alkaline phosphatase ratio combined with gamma‐glutamyl transpeptidase to aspartate aminotransferase ratio and alanine aminotransferase to aspartate aminotransferase ratio were effective diagnostic markers of alpha‐fetoprotein‐negative hepatocellular carcinoma, especially in patients with good liver function, early stage or small size.

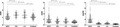

## 1 INTRODUCTION

Hepatocellular carcinoma (HCC) is one of the most common potentially lethal human malignancies worldwide.^1^ AFP (alpha‐fetoprotein) is the most widely used blood serum tumor marker for the detection of HCC. However, we found that AFP was not elevated in many HCC cases, and therefore cannot be used for broad‐based screening. Despite imaging techniques has increased the chances of HCC detection, routine ultrasonography is difficult to identify small hepatocellular nodules or benign nodules from malignant ones.^2^, ^3^ Accordingly, the diagnosis rate in patients with AFP‐NHCC was only 10.4%.^4^ Currently, many studies are looking for effective biomarkers for the diagnosis of AFP‐negative hepatocellular carcinoma (AFP‐NHCC), such as protein induced by vitamin K absence or antagonist‐II,^5^ Golgi protein 73,^6^ glypican‐3,^7^ and MicroRNAs.^8^ However, their effectiveness did not meet the clinical requirements of early diagnosis of HCC,^9^ especially for patients with AFP‐NHCC. Therefore, there is an urgent need for new biomarkers for the early diagnosis of AFP‐NHCC, ensuring the timely initiation of treatment.

Based on previous case–control studies, alanine aminotransferase (ALT), gamma‐glutamyl transferase (GGT) and aspartate aminotransferase (AST) are elevated in approximately 90% of cases diagnosed with HCC, while serum bilirubin or alkaline phosphatase (ALP) levels are elevated in more than half of cases.^10^ Although all of these single marks are related to the development of liver cancer, none of them can be used as a diagnostic or prognostic indicator of liver cancer alone. However, their ratio was reported to be related to the prognosis of HCC. It has been recently reported that the AST to ALT ratio (AAR) can indicate increased risk of early recurrence following surgical resection.^11^ Moreover, The ratio of GGT to ALP (GAPR) has a good prognostic effect on patients with large tumor size in HCC.^12^

The role of AAR and GAPR in HCC prognosis has been described in many studies. But now, there have been few reports on the diagnostic value of AAR and GAPR in AFP‐NHCC patients. There are also few reports on the diagnostic application of the GGT to AST ratio (GAR). To address this paucity of related studies, here we evaluate whether the AAR, GAR, and GAPR could serve as predictive biomarkers for AFP‐NHCC patients.

## 2 METHODS

### 2.1 Patients

From January 2014 to December 2015, there were 666 patients with AFP‐negative (<20 ng/mL) in Tongji Hospital (Tongji Medical College of Huazhong University of Science and Technology) were recruited, including 235 patients with HCC, 213 patients with chronic hepatitis (CH), and 218 patients with liver cirrhosis (LC). The inclusion criteria for patients with AFP‐Negative were as follows: (a) The clinical diagnosis was HCC, verified by histopathological examination of a surgical biopsy; Patients with CH were diagnosed with hepatitis B virus or hepatitis C virus infection for more than 6 months; LC was diagnosed by pathologic examination and typical imaging findings, including ultrasound, magnetic resonance imaging (MRI), or computed tomography (CT); (b) no organic diseases outside of the liver; (c) no infectious diseases other than HBV or HCV; (d) none of them had other types of tumors; (e) no immunity‐related diseases or hematological diseases. We also recruited 259 healthy subjects with no history of liver disease or tumor as controls. This study was approved by the Ethics Committee of Tongji Hospital (Tongji Medical College of Huazhong University of Science and Technology) with informed consent of all participants. A total of 235 patients (21–80 years old) with AFP‐NHCC were enrolled in the study, of whom 209 were HBS‐positive. Among 209 HBS‐positive AFP‐NHCC patients, 103 HBV‐DNA test value were greater than or equal to 500 IU/mL. In the CH group, 202 cases were positive for HBsAg, and the other 11 cases were positive for HCV. Of the 218 liver cirrhosis patients, 206 had an underlying hepatitis B infection, 6 had alcoholic cirrhosis, and 6 had cirrhosis due to schistosomiasis. According to the Child‐Pugh classification, 219 patients (93.2%) had level A, and 16 (6.8%) had level B disease. On the basis of the Barcelona Clinical HCC (BCLC) staging criteria, 224 cases (95.3%) were identified as stage A and the remaining 9(4.7%) as stage B.

### 2.2 Data acquisition

All data for this study were taken from each participant's electronic medical record, including gender, age, white blood cell count (WBC), blood platelet count (PLT), hemoglobin (HB), alpha‐fetoprotein (AFP), albumin (ALB), total bilirubin (TBIL), aspartate aminotransferase (AST), alkaline phosphatase (ALP), alanine aminotransferase (ALT), and gamma‐glutamyl transpeptidase (GGT). Liver function was evaluated by serum biochemical indexes, indocyanine green test, and Child‐Pugh score. The calculation formulas of AAR, GAR, and GAPR ratios were as follows: AAR = AST level/ALT level; GAR = GGT level/ AST level; GAPR = GGT level/ ALP level.

### 2.3 Statistical analysis

SPSS 20.0 statistical software was used to analyze the data. The continuous variables were compared by Mann‐Whitney U test, and the classified variables were compared by Pearson's chi‐squared test. Data that are non normally distributed are represented by medians and quartiles. The differences in laboratory parameters and clinical feature between groups were analyzed by Mann–Whitney *U*‐test or Kruskal–Wallis *H*‐test. Receiver operating characteristic (ROC) curve and area under the curve (AUC) were calculated using SPSS 20.0 statistical software and MedCalc software. Differences with a two‐sided *p* < 0.05 were considered statistically significant.

## 3 RESULTS

**FIGURE 1:**
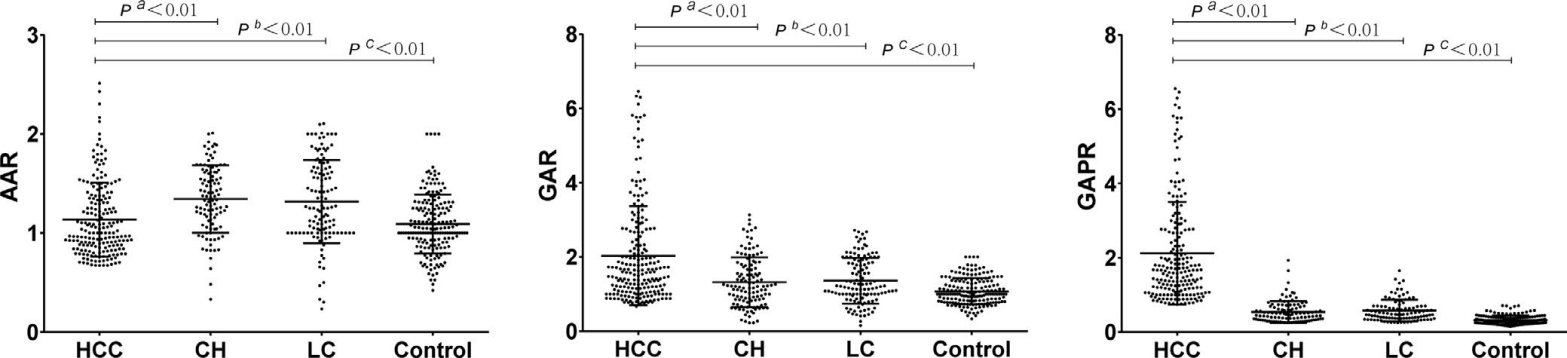
AAR, GAR and GAPR among four groups. Abbreviations: AAR, aspartate aminotransferase to alanine aminotransferase ratio; AFP, alpha‐fetoprotein; BCLC, Barcelona Clinic Liver Cancer; CH, chronic hepatitis; GAPR, gamma‐glutamyl transpeptidase to alkaline phosphatase ratio; GAR, gamma‐glutamyl transpeptidase to aspartate aminotransferase ratio; HCC, alpha‐fetoprotein‐negative hepatocellular carcinoma; LC liver cirrhosis; pa, AFP‐NHCC group vs AFP‐negative CH group; pb, AFP‐NHCC group vs AFP‐negative LC group; pc, AFP‐NHCC group vs healthy controls

The clinical characteristics of the 925 participants are shown in Table 1 and Figure 1. The clinical characteristics of 235 patients with AFP‐NHCC, 213 patients with CH, 218 patients with LC, and 259 healthy individuals (control group) were statistically summarized as medians (interquartile range) or numbers (%). According to the data, the gender of the four groups was mainly male (78.4%), and the median age of patients in the HCC group was 53 years (46–62 years). Except for Hb, other serological indexes were significantly different between the AFP‐NHCC group and the control group. The ratio of AAR, GAR, and GAPR were significantly higher in AFP‐HCC patients than in the other groups (*p* < 0.001).

**TABLE 1:** Laboratory parameters in AFP‐negative patients and healthy controls (N = 925)

Characteristics	AFP‐NHCC (N = 235)		AFP‐negative CH (N = 213)		AFP‐negative LC (N = 218)		Healthy control (N = 259)		*p* ^a^	*p* ^b^	*p* ^c^
Gender(male/female)	197/38		161/52		149/69		218/41		0.080	0.028	0.840
Age(years)	53	46–62	58	50–65	54	44–62	51	43–60	0.028	0.438	0.640
Etiology											
TBIL (μmol/L)	11.3	9.7–14.7	18.2	13.4–23.1	18.5	14.6–21.7	13.1	9.6–17.0	<0.001	<0.001	0.043
ALB (g/L)	39.6	36.6–42.3	34.9	32.8–37.0	32.7	30.8–35.0	40.8	39.6–42.8	<0.001	<0.001	<0.001
ALT (U/L)	26.0	19.0–37.0	23.0	15.0–32.0	24.0	17.0–40.0	18.0	14.0–24.0	0.038	0.585	<0.001
AST (U/L)	26.0	22.0–36.0	28.0	20.0–42.0	31.5	23.0–51.5	19.0	17.0–22.0	<0.001	<0.001	<0.001
ALP (U/L)	81.0	65.0–98.0	69.0	59.3–89.0	83.0	57.0–109.0	64.0	55.0–77.0	0.027	0.755	<0.001
GGT (U/L)	47.0	30.0–81.0	33.5	23.0–50.0	40.0	28.0–66.0	20.0	14.0–25.0	<0.001	0.360	<0.001
WBC (×10^9/^L)	5.2	4.4–6.2	3.0	2.4–4.1	3.2	2.8–3.9	6.2	5.6–6.7	<0.001	<0.001	<0.001
Hb (g/L)	126.0	121.8–130.0	96.0	82.3–113.8	89.0	84.0–100.0	126.0	123.0–131.0	<0.001	<0.001	0.612
PLT (×10^9/^L)	164.0	121.0–197.8	62.5	44.3–80.8	50.0	43.0–58.8	199.0	189.0–215.0	<0.001	<0.001	<0.001
PT (s)	14.0	13.5–14.5	16.1	15.4–16.7	16.1	15.6–16.7	14.0	13.5–14.2	<0.001	<0.001	0.792
AAR	1.21	0.91–1.46	1.35	1.1–1.61	1.33	1.06–1.66	1.02	0.88–1.29	<0.001	<0.001	<0.001
GAR	1.67	1.12–2.75	1.18	0.75–1.84	1.22	0.85–1.93	1.00	0.78–1.33	<0.001	<0.001	<0.001
GAPR	0.61	0.40–0.94	0.47	0.33–0.69	0.48	0.37–0.70	0.29	0.23–0.39	<0.001	<0.001	<0.001

Note

Data are presented as number for categorical data, median and interquartile range for nonparametrically distributed data.

Abbreviations: AAR, alanine aminotransferase to aspartate aminotransferase ratio; AFP, alpha‐fetoprotein; ALB, albumin; ALP, alkaline phosphatase; ALT, alanine aminotransferase; AST, aspartate aminotransferase; GAPR, gamma‐glutamyl transpeptidase to alkaline phosphatase ratio; GAR, gamma‐glutamyl transpeptidase to aspartate aminotransferase ratio; GGT, gamma‐glutamyl transpeptidase; Hb, hemoglobin; PLT, platelets; PT, prothrombin time; TBIL, total bilirubin; WBC, white blood cells.

^a^ AFP‐NHCC group vs AFP‐negative CH group (Kruskal‐Wallis H test).

^b^ AFP‐NHCC group vs AFP‐negative LC group (Kruskal‐Wallis H test).

^c^ AFP‐NHCC group vs healthy controls (Kruskal‐Wallis H test).

### 3.1 Correlation of AAR, GAR, and GAPR with clinical and pathologic characteristic in AFP‑NHCC

As illustrated in Table 2, both GAR and GAPR were correlated with TNM stage. GAR was associated with the Barcelona Clinic Liver Cancer (BCLC) stage. All three ratios were associated with tumor size, and patients with tumors larger than or equal to 3cm had a higher ratio than tumors smaller than 3 cm but, were not related to the cirrhosis grading or Child‐Pugh classification (*p* > 0.05 in all cases).

**TABLE 2:** Correlation of AAR, GAR, and GAPR with clinicopathological features in AFP‐NHCC (N = 235)

	n(%)		AAR		*p*	GAR		*p*	GAPR		*p*
Tumor size (cm)											
<3	67	28.5	1.01	0.88–1.34	0.029	1.45	0.62–2.17	0.037	0.49	0.34–0.79	0.007
≥3	168	71.5	1.17	0.93–1.60		1.79	0.62–2.88		0.63	0.38–0.96	
BCLC stage											
A	223	94.9	1.02	0.87–1.38	0.693	1.68	1.17–2.77	0.013	0.63	0.40–0.95	0.080
B‐C	12	5.1	1.00	0.78–1.25		1.15	0.89–1.34		0.44	0.34–0.61	
Child–Pugh classification											
A	209	88.9	1.00	0.87–1.34	0.219	1.66	1.11–2.72	0.996	0.61	0.38–0.92	0.769
B‐C	26	11.1	1.18	0.71–1.74		1.67	1.19–2.76		0.73	0.51–1.18	
TNM stage											
Ⅰ	204	86.8	1.00	0.85–1.35	0.217	1.71	1.17–2.77	0.039	0.65	0.41–0.96	0.016
Ⅱ	31	13.2	1.10	0.94–1.41		1.28	0.95–1.78		0.46	0.36–0.64	
Cirrhosis grade											
Ⅰ	150	63.8	1.00	0.86–1.35	0.554	1.63	1.18–2.41	0.905	0.61	0.38–0.93	0.282
Ⅱ	58	24.7	1.10	0.85–1.41		1.78	1.03–3.05		0.64	0.37–0.92	
Ⅲ	27	11.5	1.08	0.93–1.44		1.83	1.04–2.88		0.73	0.54–1.10	

Note

Data are presented as number (percentage) for categorical data, median (interquartile range) for nonparametrically distributed data.

Abbreviations: AAR, aspartate aminotransferase to alanine aminotransferase ratio; AFP‐NHCC, alpha‐fetoprotein‐negative hepatocellular carcinoma;BCLC, Barcelona Clinic Liver Cancer; GAPR, gamma‐glutamyl transpeptidase to alkaline phosphatase ratio; GAR, gamma‐glutamyl transpeptidase to aspartate aminotransferase ratio.

### 3.2 Logistic regression was used to differentiate AFP‐NHCC from the control group

**FIGURE 2:**
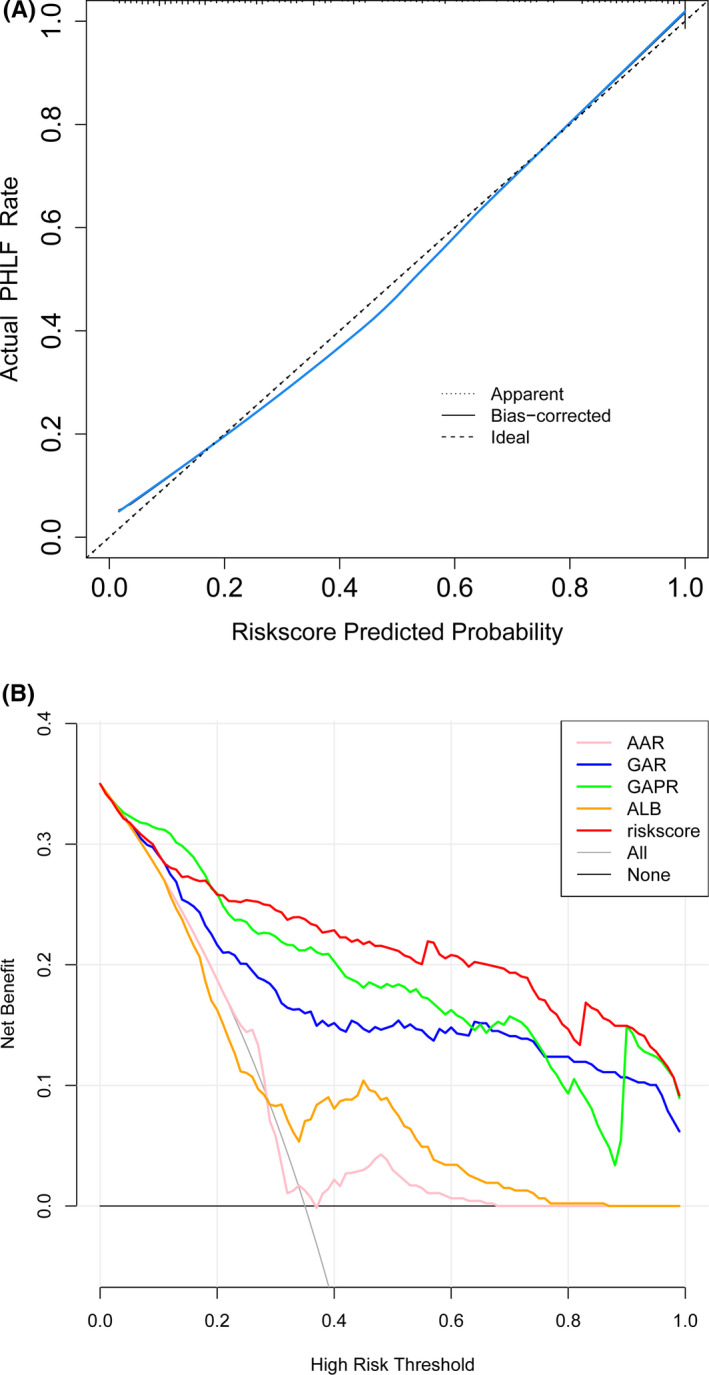
A, Calibrate curve riskscore continuous variable. B, DCA all continous variable

As illustrated in Table 3, AFP‐NHCC is associated with a number of potential risk factors, such as gender, age, HB, PLT, TBIL, ALB, AAR, GAR, and GAPR were analyzed using binary logistic regression. In order to avoid the influence of other confounding factors, multivariate analysis was performed on the variables with *p* < 0.05, and the independent effect was screened by ENTER method. Odds ratios (OR) and 95% confidence intervals (CI) for each variable were also calculated. After univariate analysis, further multivariate analysis was performed with meaningful parameters as potential independent predictors. These included ALB (OR = 0.858, 95% CI = 0.772–0.954, *p* = 0.004), AAR (OR = 4.249, 95% CI = 1.233–14.644, *p* = 0.002), GAR (OR = 2.887, 95% CI = 1.125–7.408, *p* = 0.002), and GAPR (OR = 8.229, 95% CI = 7.759–29.889, *p *< 0.001). After adjusting for these seven predictors, the results of the analysis demonstrated that ALB (*β* = −0.153, *p* < 0.001), AAR (*β* = 1.447, *p* < 0.001), GAR (*β* = 1.061, *p* < 0.001), and GAPR (*β* = 2.108, *p* < 0.001) were still important indicators closely related to the occurrence of AFP‐NHCC. The optimal model for distinguishing AFP‐NHCC patients from the control group was established through integration (logit *p *= −0.153xALB + 1.447XAAR + 1.061XGAR + 2.108XGAPR + 2.247). For this model, the AUC value was 0.905 (0.871 to 0.938). The calibration analysis the prediction model showed good agreement between the observed risk and the predicted risk (Figure 2A). The decision curve presented that if the threshold probability of a patient is >20%, the predictive value of function is better than other indexes (Figure 2B).

**TABLE 3:** Univariate and multivariate analyses used for differentiating significant predictors to distinguish AFP‐NHCC from healthy controls

Variables	Univariate analysis			Multivariate analysis		
	OR	95%CI	*p*‐value	OR	95%CI	*p*‐value
Gender	0.921	0.563–1.505	0.742			
Age(y)	1.563	0.462–3.449	0.572			
HB	0.975	0.639–1.489	<0.908			
WBC	0.629	0.523–0.755	<0.001	0.529	0.304–1.021	0.132
PLT	1.024	1.020–1.028	<0.001	0.980	0.972–1.988	0.085
TBIL	1.935	1.226–3.056	0.003	1.079	0.993–1.172	0.072
ALB	2.639	1.706–4.081	<0.001	0.858	0.772–0.954	0.004
AAR	19.179	2.550–144.239	0.012	4.249	1.233–14.644	0.002
GAR	16.752	8.321–33.724	<0.001	2.887	1.125–7.408	0.002
GAPR	16.066	9.113–28.323	<0.001	8.229	7.759–29.889	<0.001

### 3.3 Diagnostic value of AAR, GAR, and GAPR for distinguishing AFP‑NHCC from other groups

**FIGURE 3:**
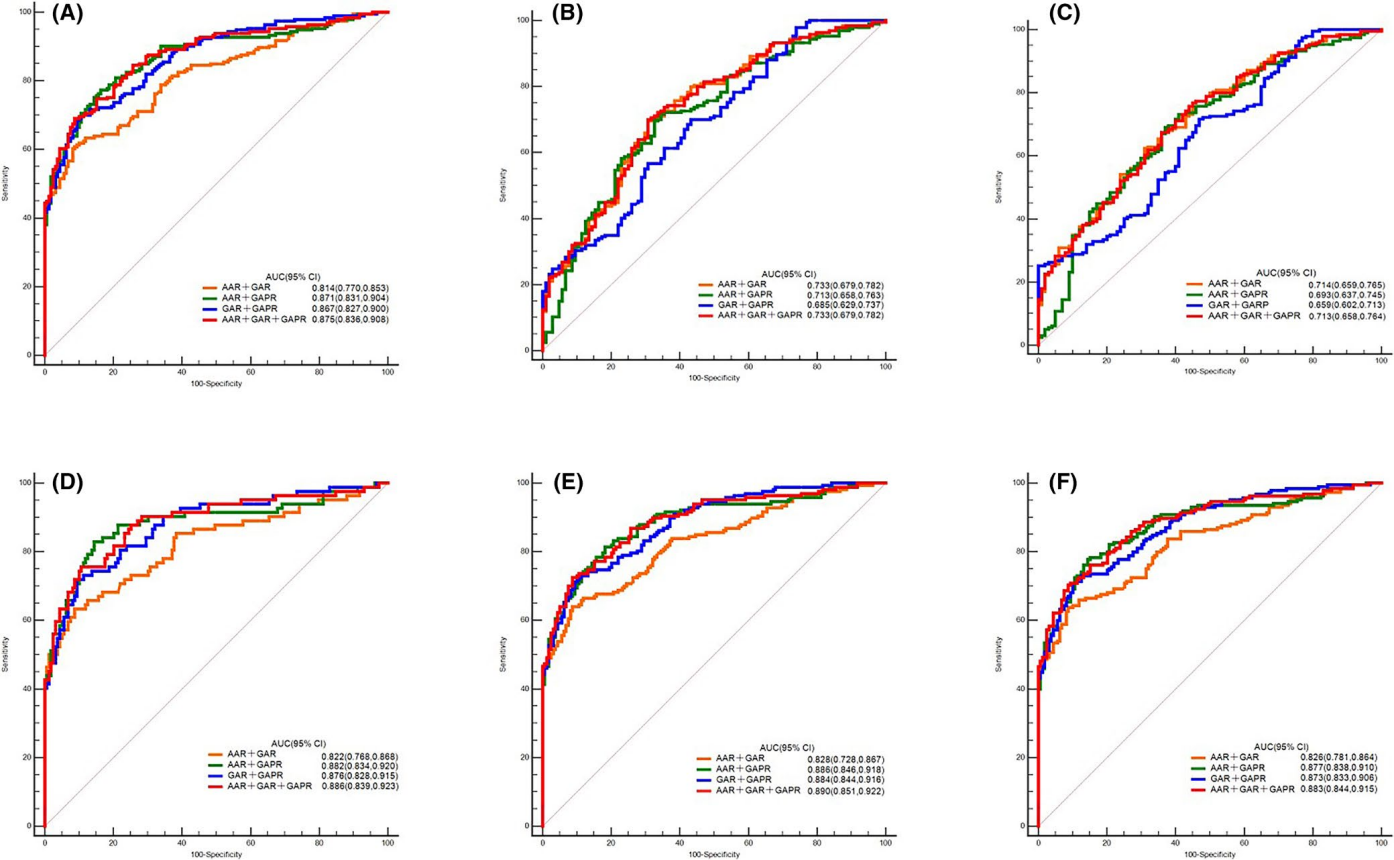
Diagnostic value of AAR, GAR and GAPR for distinguishing AFP‐NHCC from other groups. A, AFP‐NHCC patients vs healthy controls. B, AFP‐NHCC patients vs AFP‐negative CH patients. C, AFP‐NHCC patients vs AFP‐negative LC patients. D, AFP‐NHCC patients with tumor size <3 cm vs healthy controls. E, AFP‐NHCC patients with TNM‐Ⅰ stage vs healthy controls. F, AFP‐NHCC patients with BCLC‐A stage vs healthy controls. Abbreviations: AAR, aspartate aminotransferase to alanine aminotransferase ratio; AFP, alpha‐fetoprotein; AFPNHCC, alpha‐fetoprotein‐negative hepatocellular carcinoma; BCLC, Barcelona Clinic Liver Cancer; CH, chronic hepatitis; GAPR, gamma‐glutamyl transpeptidase to alkaline phosphatase ratio; GAR, gamma‐glutamyl transpeptidase to aspartate aminotransferase ratio; LC, liver cirrhosis

Table 4, Table 5, and Figure 3 show the ROC curve analysis results. Compared to healthy group, the AUC value of AAR (AUC = 0.511) did not indicate a significant difference between the two groups (*p* = 0.727). The diagnostic value of GAR (AUC = 0.804) and GAPR (AUC = 0.863) was higher, especially for AAR + GAPR (AUC = 0.871) and AAR + GAR + GAPR (AUC = 0.871), showing better diagnostic ability to distinguish AFP‐NHCC patients from the healthy control. Compared to CH, the AUC values of AAR (AUC = 0.687), GAR (AUC = 0.687), and GAPR (AUC = 0.638) were lower. The combination of AAR+GAR (AUC = 0.733) and AAR + GAR + GAPR (AUC = 0.733) showed better diagnostic ability to distinguish between AFP‐NHCC patients from CH group. Although the ratio has diagnostic value compared to LC, the combined diagnosis is not superior to the single ratio. However, the AUC value of the combination of AAR, GAR, and GAPR in AFP‐NHCC patients with TNM I stage was 0.890 (95% CI = 0.851–0.922). For those with tumors smaller than 3 cm were 0.886 (95% CI = 0.839–0.923). For patients with BCLC‐A stage disease, the value of AUC was 0.883 (95% CI = 0.844–0.915). For effective early diagnosis of AFP‐NHCC, combined use of AAR, GAR, and GAPR produced greater AUC than use alone or in pairs.

**TABLE 4:** Diagnostic value of AAR, GAR, and GAPR for distinguishing AFP‐NHCC from other groups

	Cut off	Sensitivity(%)	Specificity(%)	PV+	PV‐	LR+	LR‐	Youden index	AUC (95% CI)	*p*
AAR^a^	1.667	63.2	90.2	72.5	75.5	6.7	1.0	0.108	0.511 (0.457, 0.564)	0.727
GAR^a^	1.593	72.9	85.5	75.5	78.3	2.3	0.4	0.468	0.804 (0.759, 0.844)	<0.001
GAPR^a^	0.460	82.3	89.1	80.8	83.3	3.0	0.3	0.572	0.863 (0.823, 0.897)	<0.001
AAR^a^+GAR^a^	0.367	70.4	71.1	76.6	74.5	2.4	0.4	0.521	0.814 (0.770, 0.853)	<0.001
AAR^a^+GAPR^a^	0.498	80.5	78.8	80.7	83.1	3.8	0.3	0.610	0.871 (0.831, 0.904)	<0.001
GAR^a^+GARP^a^	0.376	83.7	84.7	81.6	80.5	3.1	0.3	0.591	0.867 (0.827, 0.900)	<0.001
AAR^a^+GAR^a^+GAPR^a^	0.395	86.8	86.2	83.3	82.2	3.4	0.3	0.603	0.875(0.836, 0.908)	<0.001
AAR^b^	1.118	40.1	89.1	50.0	67.5	1.8	0.9	0.353	0.687(0.631, 0.739)	<0.001
GAR^b^	1.238	48.8	93.2	69.7	70.9	4.2	0.8	0.263	0.687(0.631, 0.739)	<0.001
GAPR^b^	0.579	40.2	98.9	66.5	64.8	2.3	1.0	0.234	0.638(0.581, 0.693)	<0.001
AAR^b^+GAR^b^	0.623	41.3	85.5	60.5	73.1	2.9	0.7	0.388	0.733(0.679, 0.782)	<0.001
AAR^b^+GAPR^b^	0.619	49.4	86.0	60.2	72.6	2.8	0.7	0.370	0.713(0.658, 0.763)	<0.001
GAR^b^+GARP^b^	0.268	60.0	70.7	31.7	88.6	2.1	0.6	0.575	0.685(0.629, 0.737)	<0.001
AAR^b^+GAR^b^+GAPR^b^	0.612	62.3	84.5	59.4	73.2	2.7	0.7	0.393	0.733(0.679, 0.782)	<0.001
AAR^c^	1.023	27.0	92.2	53.0	68.3	2.2	0.9	0.315	0.672(0.616, 0.726)	<0.001
GAR^c^	2.719	40.0	95.8	71.4	69.9	4.8	0.8	0.253	0.658(0.600, 0.712)	<0.001
GAPR^c^	0.702	56.1	94.5	65.8	65.9	3.1	1.0	0.203	0.602(0.544, 0.658)	<0.001
AAR^c^+GAR^c^	0.362	36.0	87.1	59.0	72.5	2.8	0.7	0.315	0.714(0.659, 0.765)	<0.001
AAR^c^+GAPR^c^	0.381	33.0	89.1	61.1	72.0	3.0	0.8	0.322	0.693(0.637, 0.745)	<0.001
GAR^c^+GARP^c^	0.190	24.0	94.8	70.5	70.7	4.6	0.8	0.253	0.659(0.602, 0.713)	<0.001
AAR^c^+GAR^c^+GAPR^c^	0.414	35.0	87.6	59.3	72.3	2.8	0.7	0.318	0.713(0.658, 0.764)	<0.001

Abbreviations: AAR, aspartate aminotransferase to alanine aminotransferase ratio; AFP‐NHCC, alpha‐fetoprotein‐negative hepatocellular carcinoma; AUC, area under curve; CI, confidence interval; GAPR, gamma‐glutamyl transpeptidase to alkaline phosphatase ratio; GAR, gamma‐glutamyl transpeptidase to aspartate aminotransferase ratio; LR−, negative likelihood ratio; LR+, positive likelihood ratio; PV−, negative predictive value; PV+, positive predictive value.

^a^ AFP‐NHCC patients vs. healthy controls.

^b^ AFP‐NHCC patients vs. AFP‐negative CH patients.

^c^ AFP‐NHCC patients vs. AFP‐negative LC patients.

**TABLE 5:** Diagnostic value of AAR, GAR, and GAPR for distinguishing AFP‐NHCC from other groups

	Cut off	Sensitivity(%)	Specificity(%)	PV+	PV‐	LR+	LR‐	Youden index	AUC (95% CI)	*p*
AAR^a^	1.500	91.2	3.6	66.8	87.3	1.0	0.0	0.150	0.533(0.468, 0.597)	<0.001
GAR^a^	0.474	93.7	52.4	79.2	81.1	2.0	0.1	1.593	0.808(0.752, 0.855)	<0.001
GAPR^a^	0.448	93.0	64.6	83.6	82.8	2.6	0.1	0.612	0.873(0.824, 0.912)	<0.001
AAR^a^+GAR^a^	0.546	93.0	57.3	80.8	81.0	2.2	0.1	0.589	0.822(0.768, 0.868)	<0.001
AAR^a^+GAPR^a^	0.685	93.0	65.8	84.0	83.0	2.7	0.1	0.707	0.882(0.834, 0.920)	<0.001
GAR^a^+GARP^a^	0.592	91.8	64.6	83.4	80.3	2.6	0.1	0.625	0.876(0.828, 0.915)	<0.001
AAR^a^+GAR^a^+GAPR^a^	0.657	93.0	68.6	83.6	82.8	5.7	0.1	0.649	0.886(0.839, 0.923)	<0.001
AAR^b^	0.970	44.6	47.9	44.9	47.6	0.9	1.2	0.126	0.501(0.445, 0.556)	<0.001
GAR^b^	1.613	76.7	68.2	69.7	68.2	2.4	0.3	0.506	0.819(0.772, 0.859)	<0.001
GAPR^b^	0.508	86.1	71.8	74.4	84.5	3.1	0.2	0.601	0.880(0.840, 0.913)	<0.001
AAR^b^+GAR^b^	0.589	76.1	70.0	70.7	75.4	2.5	0.3	0.553	0.828(0.728, 0.867)	<0.001
AAR^b^+GAPR^b^	0.632	84.9	76.6	77.5	84.2	3.6	0.2	0.428	0.886(0.846, 0.918)	<0.001
GAR^b^+GARP^b^	0.577	84.2	74.8	76.1	83.3	3.3	0.2	0.618	0.884(0.844, 0.916)	<0.001
AAR^b^+GAR^b^+GAPR^b^	0.553	84.9	75.4	76.7	84.0	3.5	0.2	0.637	0.890(0.851, 0.922)	<0.001
AAR^c^	1.667	74.2	71.3	69.0	76.3	2.6	0.4	0.114	0.513(0.459, 0.567)	<0.001
GAR^c^	1.593	73.5	68.1	66.4	75.0	2.3	0.4	0.489	0.814(0.768, 0.853)	<0.001
GAPR^c^	0.460	83.6	72.4	72.2	83.7	3.0	0.2	0.584	0.869(0.828, 0.902)	<0.001
AAR^c^+GAR^c^	0.610	74.2	71.3	69.0	76.3	2.6	0.4	0.550	0.826(0.781, 0.864)	<0.001
AAR^c^+GAPR^c^	0.491	82.3	78.3	76.6	83.8	3.8	0.2	0.634	0.877(0.838, 0.910)	<0.001
GAR^c^+GARP^c^	0.580	78.6	75.1	73.0	80.3	3.2	0.3	0.608	0.873(0.833, 0.906)	<0.001
AAR^c^+GAR^c^+GAPR^c^	0.596	81.1	76.7	75.0	82.5	3.5	0.2	0.615	0.883(0.844, 0.915)	<0.001

Abbreviations: AAR, aspartate aminotransferase to alanine aminotransferase ratio; AFP‐NHCC, alpha‐fetoprotein‐negative hepatocellular carcinoma; AUC, area under curve; CI, confidence interval; GAPR, gamma‐glutamyl transpeptidase to alkaline phosphatase ratio; GAR, gamma‐glutamyl transpeptidase to aspartate aminotransferase ratio; LR−, negative likelihood ratio; LR+, positive likelihood ratio; PV−, negative predictive value; PV+, positive predictive value.

^a^ AFP‐NHCC patients with tumor size <3 cm vs. healthy controls.

^b^ AFP‐NHCC patients with TNM‐Ⅰ stage vs. healthy controls.

^c^ AFP‐NHCC patients with BCLC‐A stage vs. healthy controls.

## 4 DISCUSSION

AFP is currently the most widely used serum biomarker for HCC screening and early diagnosis, as well as the evaluation of efficacy and prognosis.^13^ However, not all HCC tumors secrete AFP, while it can also be elevated in cases of hepatitis or cirrhosis. About 30% of early HCC cases cannot be detected by AFP testing,^14^ leading to treatment delays. It is therefore important to develop new biomarkers that can identify AFP‐NHCC patients. Continued inflammatory stimulation and healing cycles within hepatocytes are thought to be a major driver in the development of HCC. However, the coexistence of cirrhosis and inflammation complicates the early diagnosis of HCC. Many inflammatory response markers have been shown to be valid, practical, and credible markers for AFP‐NHCC diagnosis and prognosis. These markers include prealbumin,^15^ D‐dimer,^15^ C‐reactive protein,^16^ and lactate dehydrogenase.^17^ Moreover, the combined application of novel molecular markers such as Golgi protein‐73 and abnormal prothrombin^18^ can improve the level of early diagnosis of HCC. However, all of these tests are expensive, and few hospitals are able to routinely carry out advanced screening, especially in primary care setting. To our best knowledge, the predictive value of AAR, GAR, and GAPR in AFP‐NHCC was unknown prior to this study, which is why we decided to investigate their diagnostic value for AFP‐NHCC, and whether their combination improve the rate of early diagnosis.

ALT testing is simple, convenient, inexpensive, and noninvasive. ALT and AST are present in the cytoplasm and mitochondria of hepatocytes, respectively. When hepatocytes were damaged, ALT and AST enter the bloodstream, leading to an increase in serum ALT and AST levels in peripheral blood. Due to the invasion of tumor in patients with HCC, normal liver cells were destroyed, serum ALT and AST increased, and the ratio of AST/ALT usually rises above one. As observed in this study, ALT and other single indicators were indeed higher in the AFP‐NHCC group than in the control group, and the difference was statistically significant (Table 1). Yang et al. stated that serum GGT combined with AST/ALT and GGT/ALT ratio had important value in the diagnosis of HCC.^18^ It has been reported that GAR is conducive to early‐stage HCC diagnosis. There are also reports that AAR is associated with the prognosis of HCC, with indications that it can be potentially used as a predictor in AFP‐NHCC. Here, we also confirmed that AAR and GAR are indeed valuable in the diagnosis of AFP‐NHCC. In previous studies, GGT and ALP were used to build different models and have been shown to be a very effectively prognostic predictor of clinical outcome in patients with HCC. Wang et al. suggested that the increase of GGT would lead to poor OS and RFS in HCC patients.^19^ Wu et al. found that when the levels of GGT, ALP, and LDH in HCC patients were low, even patients with cirrhosis also had satisfactory OS and RFS.^20^ Xu et al. suggested that preoperative serum GGT ≥115 U/L and ALP >120 U/L in HCC patients were more invasive, and their overall survival was significantly shortened.^21^ In many cancers, the increase of serum GGT is negatively correlated with survival,^22^, ^23^, ^24^ and this inverse correlation is most obvious in HCC.^25^ However, ALP is secreted in normal tissues such as the bone, liver, and small intestine and is increased when metabolic disorders, inflammation, and tumors develop.^26^ Many studies have confirmed that ALP can promote the proliferation of tumor cells, vascular invasion, and distant metastasis and also play a role in the influence of tumor formation and cell cycle changes.^27^, ^28^, ^29^ In addition, elevated serum ALP levels are common in liver disease and preoperative high serum ALP levels suggest hepatocyte damage and may be related to poor survival in HCC patients. In this study, ALP and GGT levels were significantly higher in AFP‐NHCC patients than in the control group, and the difference was statistically significant. The ratio of GGT to ALP can be regarded as a new prognostic factor in the clinical treatment of HCC.^12^ Therefore, the ratio of GGT and ALP may serve as a useful tool for predicting the occurrence of HCC.

When AAR, GAR, and GAPR were individually used for the differentiation of AFP‐NHCC patients from controls, the diagnostic value of GAPR was significantly higher than that of AAR and GAR, especially in terms of sensitivity. We also noticed that GAPR was significantly positively correlated with the tumor size in AFP‐NHCC. Although AAR was also correlated with the tumor size in AFP‐NHCC (Table 4), and there was a difference in the AUROC for each ratio (AUROC: 0.533 vs. 0.808 vs. 0.873), AAR exhibited a smaller AOC than the other ratios. In TNM stage and BCLC stage, the AUROC of GAPR and its combined ratio was larger than without it. Based on these results, we found that the ratio with combined GAPR was of higher diagnostic value than without it. In particular, the combination of three ratios had the highest diagnostic value. Therefore, we inferred that the GAPR may be more useful than AAR or GAR in the diagnosis of AFP‐NHCC. It is possible that inflammatory stimulation in or around HCC induces hepatocytes produce abundant GGT, and cancer cells themselves also synthesize GGT, further increasing the serum level of GGT than what is found in benign liver disease. Additionally, ALP also plays an important role in promoting the occurrence and development of HCC. Thus, GAPR is conducive to early‐stage HCC diagnosis. Interestingly, AAR and GAR showed a better AUC and diagnostic value in the CH and LC groups, and the combined diagnostic value was higher. Therefore, the single ratio may not be sufficient to explain its diagnostic significance, while the combined multi‐index diagnosis may have a higher positive value.

Child‐Pugh grades, and tumor size were closely related to HCC.^30^, ^31^, ^32^ Songlin et al^33^ revealed the association between AFP and clinicopathological features of HCC, including Child‐Pugh grades and tumor size, which was consistent with our findings. In other studies, the relationship between hematologic parameters (GAR and GAPR) and clinical characteristics (Child‐Pugh grades and tumor size) have been demonstrated.^22^, ^23^ Study has also demonstrated that the GAPR was significant associated with BCLC stage, vascular invasion, and tumor size of HCC.^12^ GAR was a superior prognostic factor for survival outcome than several potential prognostic indices.^34^ Because these indicators are related to the occurrence and development of HCC, we select them as diagnostic indicators for APF‐NHCC. This study is the first to evaluate the effectiveness of AAR combined with GAR and GAPR in the diagnosis of AFP‐NHCC. AAR combined GAR, GAPR in tumors less than 3 cm, BCLC‐A stage and TNMⅠstage of APF‐NHCC patients also have favorable diagnostic value.

This study manifested that AAP, GAR, and GAPR may be valuable indicators for evaluating AFP‐NHCC patients. AAR, GAR, and GAPR had better AUC values and sensitivity in differentiating AFP‐NHCC patients from control group, whereby the combination of their ratios showed a higher diagnostic value. Accordingly, numbers of studies have also found biomarkers that could help diagnose AFP‐NHCC. when Golgi protein 73,^35^ Alpha‐fetoprotein‐L3,^35^ and Des‐Gamma‐Carboxy Prothrombin^36^ were used to distinguish AFP‐NHCC from controls, the corresponding AUC values were 0.7811, 0.6094, and 0.856, while the sensitivity was 66%, 50%, and 76.3%, respectively. The AUC values were significantly lower than that of the combination of AAP, GAR, and GAPR and the latter showed superior sensitivity in examining AFP‐NHCC. Moreover, the AAR combination with GAR and GAPR had a greater AUC than either of them or a combination of two for discriminating AFP‐NHCC patients from the other groups. One retrospective study by Zhang et al.^35^ showed that the detection rate of DCP combined with AFP‐L3 in AFP‐NHCC patients was only 68.4%, which is lower than what we obtained by combining the AAR, GAR, and GAPR. Similarly, Zhang et al.^31^ showed that the sensitivity of AFP‐L3 combined with GP73 in the detection of liver cancer was 40%, which was much lower than the detection results of AAR combined with GAR and GAPR. In addition, AAR, GAR, and GAPR were also moderately good markers for differentiating AFP‐NHCC patients from AFP‐negative CH and AFP‐negative LC groups. Therefore, the present outcome suggested that the combined application of AAR, GAR, and GAPR can improve the efficiency of clinical diagnosis by distinguishing AFP‐NHCC from other groups.

Nevertheless, there are some limitations should be noted in interpreting the results of this study.

The AFP negative cases were all from the same hospital and in a limited numbers, and may have skewed the predictive value of these markers. Secondly, most cases of AFP‐NHCC were positive for hepatitis B antigen, which may not represent all types of AFP‐NHCC. Therefore, future prospective studies require a multi‐center approach, large sample size and different types of AFP‐NHCC cases to verify the results.

## 5 CONCLUSIONS

This study showed that AAR, GAR, and GAPR can be used as diagnostic indexes of AFP‐NHCC. AAR combination with GAR and GAPR has great potential as an economical, simple, and effective diagnostic test for patients with AFP‐NHCC, especially those with good liver function and early or small AFP‐NHCC tumors.

## ETHICS APPROVAL AND CONSENT TO PARTICIPATE

This study was conducted in accordance with the standards of the Declaration of Helsinki and approved by the medical ethics committee of Tongji Hospital, Huazhong University of Science and Technology, China. Written informed consent for hepatectomy and further research was obtained from all patients.

## CONFLICT OF INTEREST

The authors have stated explicitly that there are no conflicts of interest in connection with this article.

## AUTHORS CONTRIBUTIONS

Zhiyong Huang contributed to the study design. Jiang Li, Haisu Tao, and Erlei Zhang contributed to the collection of data. Jiang Li and Haisu Tao contributed to the analysis and interpretation of data. Jiang Li and Zhiyong Huang contributed to the writing of the article. Zhiyong Huang and Erlei Zhang contributed to the financial support. Zhiyong Huang and Erlei Zhang contributed to the revision of the article and statistical analysis. All authors read and approved the final version of the article.

## Data Availability

The data that support the finding of this study are available from the corresponding author upon reasonable request.
